# Reduced viral burden in paralytic compared to furious canine rabies is associated with prominent inflammation at the brainstem level

**DOI:** 10.1186/1746-6148-9-31

**Published:** 2013-02-14

**Authors:** Shanop Shuangshoti, Nischol Thepa, Pornchai Phukpattaranont, Akanitt Jittmittraphap, Nirun Intarut, Veera Tepsumethanon, Supaporn Wacharapluesadee, Paul Scott Thorner, Thiravat Hemachudha

**Affiliations:** 1 Department of Pathology, Faculty of Medicine, Chulalongkorn University, Bangkok, Thailand; 2 Chulalongkorn GenePRO Center, Faculty of Medicine, Chulalongkorn University, Bangkok, Thailand; 3 Department of Clinical Pathology, Faculty of Medicine, Chulalongkorn University, Bangkok, Thailand; 4 Department of Electrical Engineering, Faculty of Engineering, Prince of Songkla University, Songkhla, Thailand; 5 WHO Collaborating Center for Research and Training on Viral Zoonoses, Bangkok, Thailand; 6 Clinical Research Center, Faculty of Medicine, Chulalongkorn University, Bangkok, Thailand; 7 Queen Saovabha Memorial Institute, Bangkok, Thailand; 8 Department of Medicine, Faculty of Medicine, Chulalongkorn University, Bangkok, Thailand; 9 Department of Laboratory Medicine, Hospital for Sick Children and University of Toronto, Toronto, Canada

**Keywords:** Rabies, Furious rabies, Paralytic rabies, Rabies viral antigen, Inflammation

## Abstract

**Background:**

The mechanisms that differentiate rabies infections into furious and paralytic forms remain undetermined. There are no neuropathological features in human brains that distinguish furious and paralytic rabies. This could be due to methodology and/or examination of specimens late in the disease course.

In this study, postmortem examination of brain (5 furious and 5 paralytic) and spinal cord (3 furious and 3 paralytic) specimens was performed in 10 rabies-infected dogs, sacrificed shortly after developing the illness. Rabies virus (RABV) antigen (percentage of positive neurons, average antigen area in positive neurons and average antigen area per neuron) and RNA were quantified at 15 different central nervous system (CNS) regions. The distribution and degree of inflammation were also studied.

**Results:**

More RABV antigen was detected in furious rabies than paralytic in many of the CNS regions studied. Caudal-rostral polarity of viral antigen distribution was found in both clinical forms in order from greatest to least: spinal cord, brainstem, cerebellum, midline structures (caudate, thalamus), hippocampus, and cerebrum. In contrast, RABV RNA was most abundant in the cerebral midline structures. Viral RNA was found at significantly higher levels in the cerebral cortex, thalamus, midbrain and medulla of dogs with the furious subtype. The RNA levels in the spinal cord were comparable in both clinical forms. A striking inflammatory response was found in paralytic rabies in the brainstem.

**Conclusions:**

These observations provide preliminary evidence that RABV antigen and RNA levels are higher in the cerebrum in furious rabies compared to the paralytic form. In addition, brainstem inflammation, more pronounced in paralytic rabies, may impede viral propagation towards the cerebral hemispheres.

## Background

Rabies is an infectious disease of the central nervous system (CNS) caused by a neurotropic RNA virus in the family *Rhabdoviridae*, genus *Lyssavirus*[1]. The worldwide number of rabies deaths estimated by the World Health Organization survey in 1998 was 55,000 annually with the highest incidence in Asia [2] and this number increased to 70,000 in 2011 [3]. Rabies encephalitis is almost universally fatal, with only six survivors having been recorded to date [4-7]. In Thailand, 10–20 patients (0.03 per 100,000 populations) die of rabies each year [8].

Rabies can manifest as furious or paralytic forms in humans and dogs. Limbic signs dominate the clinical picture in the former whereas a paralysis of lower motor neuron type dominates the latter [9]. The ratio between furious and paralytic rabies in human cases is approximately 3:1 [10]. Furious rabies patients tend to have a shorter survival (average 5.7 days compared to 11 days in the paralytic form) [9,11]. The mechanisms that result in two distinct clinical forms remain enigmatic. In human cases, no differences were found in rabies virus (RABV) antigen distribution or the degree of inflammation between furious and paralytic rabies cases at post-mortem [12]. RABV antigen was confined mainly to midline structures (thalamus, brainstem and basal ganglia) in patients who died earlier than 5 days after clinical onset. Several recent clinical, electrophysiological, and neuropathological findings have, however, suggested possible different mechanisms for the furious and paralytic forms of rabies. Peripheral nerve dysfunction, axon- or myelinopathy, is considered to be responsible for the clinical weakness in paralytic rabies [11]. In contrast, in the case of furious rabies, dysfunction of spinal cord anterior horn cells has been identified by electrophysiology, and central chromatolysis by postmortem examination, despite an absence of weakness [9].

Although previous magnetic resonance imaging (MRI) studies in human rabies patients have revealed no differences between the two clinical forms [13], diffusion tensor imaging technique has demonstrated disruption of neural tract integrity at the brainstem level in dogs with the paralytic form of rabies [14]. A marked decrease in the mean diffusivity values, representing cytotoxic edema, was noted in the cerebral hemispheres without evidence of blood brain barrier leakage in paralytic dogs [14]. The blood brain barrier was also intact in furious dogs but with minimal cytotoxic brain edema.

Discrepant MRI results between rabies in humans and dogs may be explained by timing of examination, which is late in the course of disease in humans. The current study was, therefore, conducted in rabies-infected dogs, sacrificed shortly after the onset of symptoms. The current study included a neuropathological assessment of the brain and spinal cord, including a determination of the distribution and amount of RABV antigen and inflammatory response. These results were then correlated with antemortem imaging studies. A significantly greater amount of viral antigen was found in many regions of the brain and spinal cord in furious compared to paralytic rabies. This was also true for RABV RNA at regions above the level of the spinal cord. Inflammation was more prominent in the brainstem in paralytic rabies suggesting the inflammatory response may be critical in influencing clinical presentation and disease course.

## Methods

### Animals

The selected animals were community dogs with no particular owner and were fed by local people. Such individuals brought these animals to the Queen Saovabha Memorial Institute (QSMI), Bangkok, Thailand, for observation after they had bitten someone. These animals displayed no abnormal signs when first brought in. None had a history of vaccination. Once the animals started having abnormal signs, saliva samples were sent for rabies confirmation by nucleic acid sequence–based amplification (NASBA) as previously described [15]. While waiting for NASBA results, which usually took 3–4 hours, these animals were closely observed by a veterinarian experienced in rabies infection [VT] for signs indicative of furious (aggression, biting/attacking the cage on seeing people approaching) or paralytic rabies (no or very minimal aggression with dropped jaw and hind limb paresis) [16]. All animals that exhibited definite signs of either furious or paralytic rabies were humanely sacrificed as soon as the diagnosis was confirmed. None of them showed ambiguous clinical signs or changed from the furious to the paralytic form or *vice versa*. The duration between the first abnormal sign and autopsy in these animals was approximately 24 hours. Animals that were scheduled for MRI study were sedated prior to MRI examination, which was done within 24 hours after the first clinical sign. This procedure, including transfer to the MRI facility, MRI examination, and euthanasia took 4–6 hours, making the interval between the first clinical signs to autopsy in MRI-examined dogs approximately 36 hours. The period within 36 hours between the first sign and euthanasia for all animals in the study was based on the observation of 957 confirmed rabid dogs (two-third were furious) that were quarantined until death [17]. The median survival time of rabid dogs was 4 days, with 25% of them died within 48 hours, and they usually lapsed into coma 12 hours before death.

During the 3 years of the specimen collection phase, there were 124 dogs observed at the Quarantine and Diagnostic Unit of the QSMI and 41 of them were determined to have rabies infection. 10 rabid dogs (5 furious and 5 paralytic) that met the criteria set above were considered to be in the early stage of the disease. All animals were conscious prior to euthanasia, except for the 4 (2 furious and 2 paralytic) that were sedated for imaging purposes. The results of the MRI studies have been previously published [18]. Brains were available for pathological examination in all 10 dogs and spinal cords in 6 dogs (3 furious and 3 paralytic).

### Collection of samples

The diagnosis of rabies was confirmed by the presence of rabies antigen and viral nucleocapsid RNA in the brain by a direct fluorescent antibody test and by NASBA [15]. All tissues were initially stored at −80°C immediately upon sacrifice. Samples for histology were taken immediately after thawing, fixed in 10% buffered formalin, routinely processed and embedded in paraffin wax. Brain and spinal cord tissue was collected from 11 sites in the brain and 4 in the spinal cord. A canine brain transaction atlas [19] was used to identify the region of interest as follows: frontal tip, parietal lobe (level 2), temporal lobe (level 5), occipital tip, cerebellum (vermis), caudate nucleus (level 2), hippocampus (including CA1–4 regions) (level 5), thalamus (level 4), midbrain (level 7), pons (level 12), medulla (level 17), and spinal cord (cervical, thoracic, lumbar, and sacral levels). For histologic examination, one section was taken from each brain and brainstem region and 3 from each spinal cord level. Samples from the 11 brain regions and from each spinal cord level were submitted for RABV RNA study as described below.

### RABV quantification by TaqMan real-time PCR

Extraction of RNA, reverse transcription, and RABV quantification by TaqMan real-time polymerase chain reaction (PCR) was done as previously described [18]. The rabies viral load (N gene) was expressed as the copy number of rabies per *μ*g total RNA. The levels of viral RNA in the different brain regions in 8 dogs (4 of each clinical form) have previously been published [18]. In the present study, 2 more cases (one of each clinical form) and spinal cord tissue (6 cases, 3 of each clinical form) were included for analysis.

### Immunohistochemical studies for rabies viral antigen and inflammatory cells

Indirect immunoperoxidase staining was carried out on four-micrometer-thick sections using an automated stainer (Ventana Benchmark LT, Tucson, USA). All tissue sections were cut by the same technologist using the same microtome. All steps of the immunostaining procedure were performed in a closed system using identical settings between runs, in order to maintain consistency in immunostaining. This system includes blocking with normal serum of the same species as the primary antibody, prior to application of the primary antibody. Primary antibodies included a polyclonal anti-rabies nucleocapsid antibody (Bio-Rad; Marnes-la-Coquette, France) at a dilution of 1:80, a polyclonal anti-CD3 antibody (DakoCytomation; Glostrup, Denmark at a dilution of 1:200), and a polyclonal anti-CD20 antibody (Abnova; CA, USA at a dilution of 1:100). Both CD3 and CD20 antibodies are directed against human epitopes but cross react some other mammalian species including dog. Although the anti-rabies nucleocapsid antibody was conjugated to FITC, fluorescence was not used for detection, and a secondary reagent was required for signal detection by light microscopy. The other two primary antibodies were unconjugated. The detection is done by the Ventana Ultraview technology that uses a multimeric secondary reagent. The negative control for each antibody consisted of omission of the primary antibody.

### Quantitative analysis of rabies viral antigen

Semiquantitative analysis was done on the sections immunostained for rabies antigen. The amount of rabies antigen in neurons for each anatomical site was scored by a pathologist [SS] on a scale of 0 to 4+ as follows: 0 = negative, 1+ = 1-25% positive neurons, 2+ = 26-50%, 3+ = 51-75%, and 4+ > 75%. Analysis was performed without knowledge of the clinical data.

**Figure 1:**
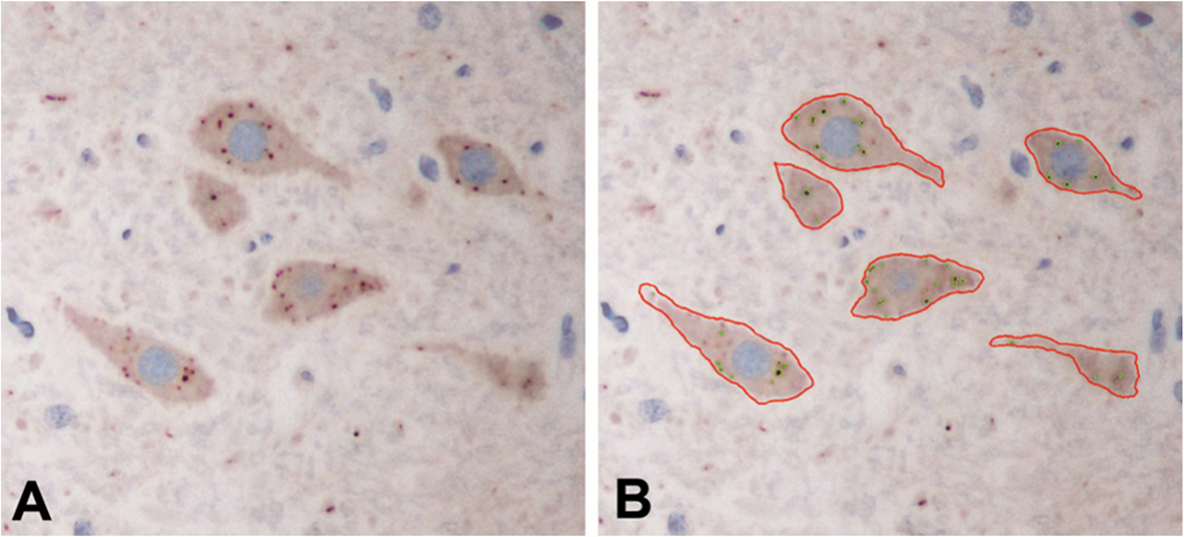
**Quantitative analysis of rabies viral antigen.** Following immunostaining for rabies antigen (**A**), all neurons in each captured image are manually outlined (**B**) and analyzed by computer software. The three antigen parameters assessed are: (1) percentage of positive neurons, (2) average antigen area in positive neuron, and (3) average antigen area per neuron. (**A** and **B**, immunoperoxidase, original magnification X400).

Quantitative analysis was performed using the immunostained slides for rabies antigen, which were captured in color by a digital camera (Nikon DXM1200F) attached to a microscope (Nikon ECLIPSE 80i) (Nikon Instech Co., Ltd., Japan) at 400× magnification. For each slide of the cerebral cortices, caudate nucleus and thalamus; the area with maximum RABV antigen was selected and neurons were digitally captured. Neurons in all layers of the cerebral cortices were included for analysis. Consecutive fields of the CA1 through CA4 regions were selected for the hippocampus, and the Purkinje cell layer for the cerebellum. All groups of neurons in the brainstem were captured, and only the gray matter was selected for all spinal cord levels. Each field covered an area of 0.0705 mm^2^ and from 10 to 20 fields (0.7-1.41 mm^2^) were captured from each slide. All neurons in each field were manually outlined using Adobe Photoshop. Quantitative assessment was performed by a computer-aided system (Cell Image Analyzer) (Figure 1) as previously described [20]. This system measures the amount of chromogen signal regardless of the pattern of staining (e.g., granular vs. diffuse). Parameters assessed included: (1) percentage of positive neurons, (2) average antigen-positive area in positive neurons, and (3) average antigen-positive area per neuron [parameter 1 × parameter 2 ÷ 100]. The first parameter indicates how many neurons are affected while the others indicate the extent of involvement. The average number of neurons counted per dog was: cerebral cortices, 1881.6; cerebral midline (caudate and thalamus) and hippocampus, 782.6; cerebellum, 61.9; brainstem, 382.2; and spinal cord, 565.4.

### Quantitative analysis of inflammatory response

Semi-quantitative analysis was performed on four-micrometer-thick sections of all paraffin blocks stained with hematoxylin and eosin. The inflammatory response in each anatomical site was scored by a pathologist [SS] on a scale of 0 to 3+ as follows: 0 = no inflammation, 1 = mild inflammation (mild microglial activation without formation of microglial nodules and/or mild perivascular lymphocytic cuffing), 2 = moderate inflammation (microglial activation with formation of microglial nodules and/or moderate lymphocytic perivascular cuffing), and 3 = marked inflammation (microglial activation with formation of microglial nodules with extension of microglia aggregates outside nodules, and/or marked perivascular lymphocytic cuffing with aggregates of lymphocytes outside the perivascular area). The degree of perivascular lymphocytic cuffing was defined by the number of layers of lymphocytes around vessels: mild, 1–2 cells; moderate, 3–4 cells, and marked, 5 cells or more.

Quantitative analysis was done on all CD3-immunostained slides. Images were digitally captured in and around the area of perivascular lymphocytic cuffing. 10 to 20 fields (0.7-1.41 mm^2^) were captured from each slide. Positive cells were manually counted, and the density of positive cells (the number of cell per 0.0705 mm^2^) calculated. CD20-immunostained slides were not quantitated since very few positive cells were found. The assessments were done without knowledge of the clinical data.

### Statistical analysis

In quantitative assays, data from all fields were used to generate the average and SD. RABV antigen parameters and amounts of RNA were compared within the same group of animals at different CNS regions and between the different groups (furious versus paralytic), using the Mann–Whitney U test for a one-tailed test. Density of T cells was also compared between the different groups. A one-sided test was used because the semiquantitative analyses showed a trend towards a greater antigen burden in furious rabies (see Table 1) and towards greater inflammation in paralytic rabies (see Table 2). Statistical analyses were performed using Statistical Package for Social Science software (SPSS version 17.0, SPSS Inc., Chicago, IL, USA). A result was considered statistically significant at *p* < 0.05.

**Table 1:** Semiquantitative analysis of rabies viral antigen in canine paralytic and furious rabies

	**Positive neurons**									
	**Paralytic rabies**					**Furious rabies**				
	**1**	**2**	**3**	**4**	**5**	**1**	**2**	**3**	**4**	**5**
Frontal cortex	0	1+	1+	1+	2+	1+	1+	2+	2+	3+
Parietal cortex	0	1+	2+	2+	2+	1+	2+	2+	3+	3+
Temporal cortex	0	1+	1+	2+	3+	1+	1+	2+	2+	3+
Occipital cortex	0	0	1+	1+	1+	1+	2+	1+	2+	3+
Hippocampus	0	0	2+	3+	4+	3+	3+	2+	3+	4+
Caudate	0	1+	1+	2+	3+	2+	2+	2+	2+	3+
Thalamus	0	2+	1+	3+	3+	1+	2+	4+	NA	4+
Cerebellum	0	3+	3+	3+	3+	3+	3+	3+	4+	4+
Midbrain	0	3+	4+	4+	4+	4+	4+	4+	3+	4+
Pons	0	4+	2+	4+	4+	2+	4+	4+	2+	4+
Medulla	0	3+	3+	4+	4+	NA	4+	4+	4+	4+
Cervical cord	3+	4+	NA	4+	NA	NA	NA	4+	4+	4+
Thoracic cord	2+	4+	NA	4+	NA	NA	NA	4+	4+	4+
Lumbar cord	3+	3+	NA	4+	NA	NA	NA	4+	4+	4+
Sacral cord	3+	3+	NA	3+	NA	NA	NA	4+	4+	4+

NA = Not available.

**Table 2:** Semiquantitative analysis of inflammation in canine paralytic and furious rabies

	**Inflammation**									
	**Paralytic rabies**					**Furious rabies**				
	**1**	**2**	**3**	**4**	**5**	**1**	**2**	**3**	**4**	**5**
Frontal cortex	1+	1+	1+	1+	1+	1+	0	1+	1+	1+
Parietal cortex	1+	1+	1+	1+	0	1+	0	1+	1+	1+
Temporal cortex	1+	1+	1+	1+	1+	1+	0	1+	1+	1+
Occipital cortex	1+	1+	1+	1+	0	1+	0	1+	1+	1+
Hippocampus	1+	1+	1+	1+	1+	1+	0	1+	1+	1+
Caudate	1+	1+	1+	1+	1+	1+	0	1+	1+	1+
Thalamus	1+	1+	1+	1+	1+	1+	1+	1+	NA	1+
Cerebellum	0	1+	1+	1+	1+	0	1+	1+	1+	1+
Midbrain	3+	3+	3+	2+	2+	1+	1+	1+	1+	1+
Pons	3+	2+	3+	2+	2+	1+	1+	2+	1+	1+
Medulla	3+	3+	3+	2+	2+	NA	1+	2+	1+	1+
Cervical cord	1+	1+	NA	1+	NA	NA	NA	1+	0	1+
Thoracic cord	1+	1+	NA	1+	NA	NA	NA	1+	0	1+
Lumbar cord	0	1+	NA	1+	NA	NA	NA	0	0	0
Sacral cord	0	0	NA	0	NA	NA	NA	0	0	1+

NA = Not available.

## Results

### Patterns of RABV antigen, RABV RNA, and inflammation in paralytic rabies

**Figure 2:**
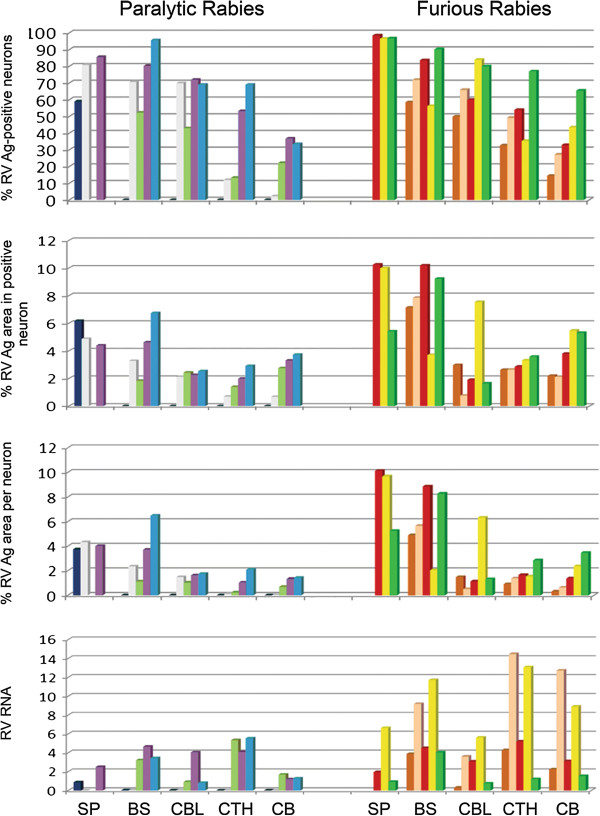
**Rabies viral antigen and RNA pattern in paralytic and furious rabies.** Rabies antigen parameters and RNA levels are shown for the major CNS regions. Each column color represents a single animal. Note that rabies viral antigen and RNA in one paralytic dog (dark blue column) was found exclusively in the spinal cord. RABV RNA is expressed in copies/*μ*g total RNA × 10^8^. RABV = rabies virus; Ag = Antigen; SP = spinal cord; BS = brainstem; CBL = cerebellum; CTH = caudate, thalamus, and hippocampus; and CB = cerebrum. No spinal cord tissue was available for paralytic rabies cases # 3 and 5 and furious rabies cases # 1 and 2 (refer to SP bars).

**Figure 3:**
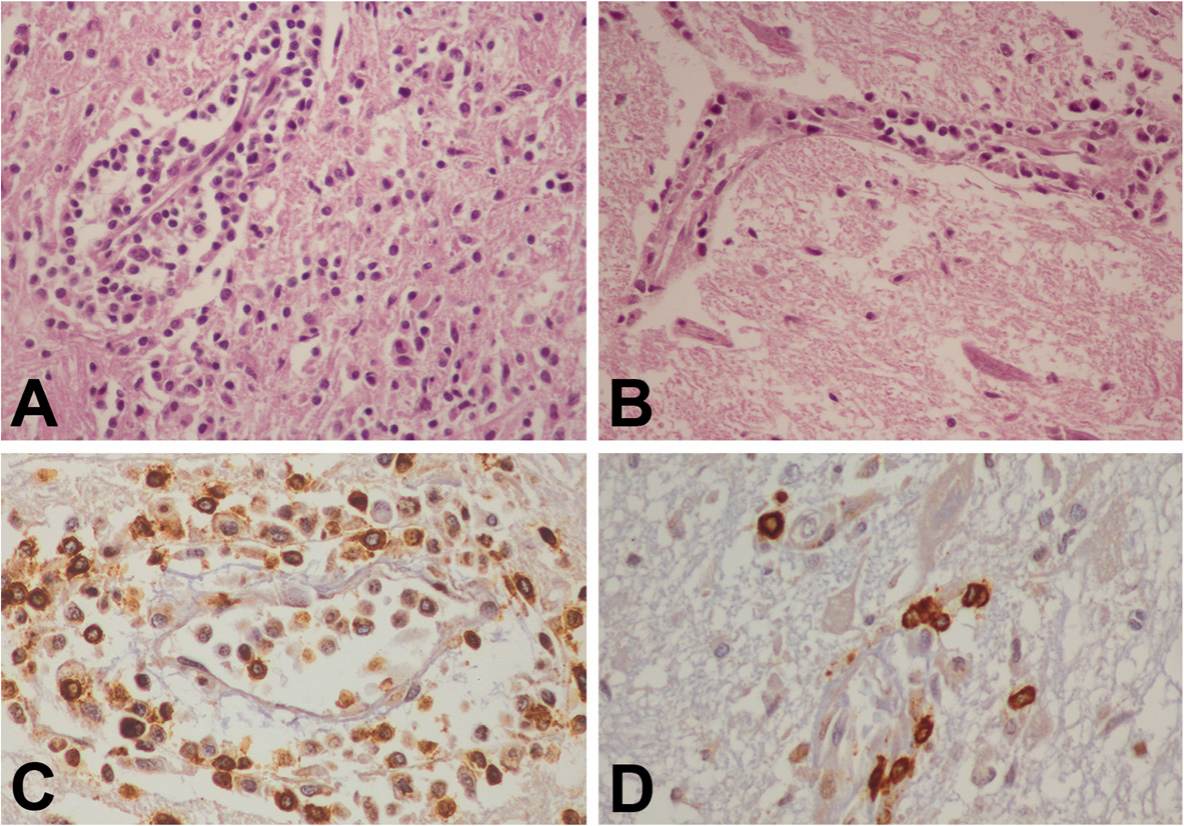
**Brainstem inflammation in canine rabies.** Striking inflammation (**A**) with a dense T cell infiltrate (**C**) in brainstem of paralytic rabies is shown, in comparison with a mild inflammatory response (**B**) and fewer T cells (**D**) in furious rabies. (**A** and **B**, hematoxylin and eosin stain, original magnification X 400; **C** and **D**, CD3 immunostain, original magnification X 400).

The semiquantitative results for number of neurons positive for RABV antigen are presented in Table 1, and the quantitative results for viral antigen parameters RNA amount according to major CNS regions of individual dogs are summarized in Table 3 and depicted in Figure 2. Both semiquantitative and quantitative studies show similar trends. RABV antigen-positive neurons at CNS regions were found most abundantly in the spinal cord followed by brainstem, cerebellum, cerebral midline structures (caudate, thalamus), hippocampus, and cerebrum. A significantly greater percentage of RABV-positive neurons was found in the spinal cord, as compared to the cerebrum and cerebral midline structures. The same trend was observed with the other viral antigen parameters (average antigen-positive area in positive neurons and average antigen-positive area per neuron). The largest quantities of viral mRNA were, however, observed in the cerebral midline structures and hippocampus, followed by brainstem. There was no significant difference in viral RNA levels between the spinal cord, brainstem, cerebellum, thalamus-caudate, hippocampus, and cerebrum. Prominent inflammation was noted in the brainstem (Table 2 and Figure 3).

**Table 3:** Comparison of rabies viral antigen and RNA in major CNS regions in canine paralytic rabies

	**Spinal cord**		**Brainstem**		**Cerebellum**		**Cerebral midline**		**Cerebrum**	
	**(Mean ± SD)**	***P value***	**(Mean ± SD)**	***P value***	**(Mean ± SD)**	***P value***	**(Mean ± SD)**	***P value***	**(Mean ± SD)**	***P value***
***% RABV antigen-positive neurons***										
Spinal cord	75.19 ± 14.14			0.228		0.090		0.026		0.012
Brainstem		0.228	59.8 ± 36.94			0.201		0.104		0.047
Cerebellum		0.090		0.201	50.8 ± 30.78			0.124		0.047
Cerebral midline		0.026		0.104		0.124	29.53 ± 29.86			0.337
Cerebrum		0.012		0.047		0.047		0.337	18.95 ± 17.16	
***% RABV antigen area in positive neuron***										
Spinal cord	5.14 ± 0.92			0.148		0.013		0.013		0.013
Brainstem		0.148	3.29 ± 2.58			0.201		0.104		0.265
Cerebellum		0.013		0.201	1.87 ± 1.06			0.201		0.201
Cerebral midline		0.013		0.104		0.201	1.39 ± 1.13			0.265
Cerebrum		0.013		0.265		0.201		0.265	2.09 ± 1.65	
***% RABV antigen-positive area per neuron***										
Spinal cord	4.03 ± 0.28			0.089		0.013		0.013		0.013
Brainstem		0.089	2.74 ± 2.51			0.147		0.071		0.104
Cerebellum		0.013		0.147	1.18 ± 0.71			0.201		0.104
Cerebral midline		0.013		0.071		0.201	0.69 ± 0.88			0.500
Cerebrum		0.013		0.104		0.104		0.500	0.70 ± 0.69	
***RABV RNA*** (copies/*μ*g total RNA) × 10^8^										
Spinal cord	1.12 ± 1.26			0.148		0.441		0.228		0.441
Brainstem		0.148	2.27 ± 2.11			0.174		0.377		0.087
Cerebellum		0.441		0.174	1.52 ± 1.67			0.174		0.458
Cerebral midline		0.228		0.377		0.174	2.99 ± 2.78			0.125
Cerebrum		0.441		0.087		0.458		0.125	0.83 ± 0.77	

Underlined *P* values indicate significant difference.

### Patterns of RABV antigen, RABV RNA, and inflammation in furious rabies

The semiquantitative results for the number of neurons positive for RABV antigen are presented in Table 1, and the quantitative results for viral antigen parameters and RNA amount according to major CNS regions of individual dogs are summarized in Table 4 and depicted in Figure 2. Similar to paralytic rabies, the spinal cord contained the most numerous RABV antigen-positive neurons, followed by brainstem, cerebellum, cerebral midline structures (caudate, thalamus), hippocampus, and cerebrum (Table 4). The percentage of RABV-positive neurons in the spinal cord was significantly greater than in the brainstem and brain regions. A similar pattern was noted with all three antigen parameters. As with paralytic rabies, the largest quantities of viral RNA were observed in the cerebral midline structures and hippocampus, followed by brainstem. Viral RNA level in the brainstem was significantly greater than in the cerebellum. Inflammation was generally mild throughout the entire neuraxis (Table 2).

**Table 4:** Comparison of rabies viral antigen and RNA in major CNS regions in canine furious rabies

	**Spinal cord**		**Brainstem**		**Cerebellum**		**Cerebral midline**		**Cerebrum**	
	**(Mean ± SD)**	***P value***	**(Mean ± SD)**	***P value***	**(Mean ± SD)**	***P value***	**(Mean ± SD)**	***P value***	**(Mean ± SD)**	***P value***
***% RABV antigen-positive neurons***										
Spinal cord	97.32 ± 1.09			0.013		0.013		0.013		0.013
Brainstem		0.013	72.17 ± 14.99			0.377		0.024		0.014
Cerebellum		0.013		0.377	68.0 ± 14.07			0.038		0.014
Cerebral midline		0.013		0.024		0.038	49.69 ± 17.72			0.125
Cerebrum		0.013		0.014		0.014		0.125	36.75 ± 19.18	
***% RABV antigen area in positive neuron***										
Spinal cord	8.55 ± 2.7			0.228		0.026		0.013		0.026
Brainstem		0.228	7.62 ± 2.49			0.014		0.004		0.023
Cerebellum		0.026		0.014	2.97 ± 2.67			0.174		0.125
Cerebral midline		0.013		0.004		0.174	3.01 ± 0.43			0.301
Cerebrum		0.026		0.024		0.125		0.301	3.77 ± 1.62	
***% RABV antigen-positive area per neuron***										
Spinal cord	8.35 ± 2.7			0.089		0.026		0.013		0.013
Brainstem		0.089	5.95 ± 2.75			0.024		0.008		0.014
Cerebellum		0.026		0.024	2.16 ± 2.36			0.232		0.458
Cerebral midline		0.013		0.008		0.232	1.67 ± 0.72			0.338
Cerebrum		0.013		0.014		0.458		0.338	1.63 ± 1.29	
***RABV RNA*** (copies/*μ*g total RNA) × 10^8^										
Spinal cord	3.16 ± 3.04			0.089		0.327		0.148		0.148
Brainstem		0.089	6.66 ± 3.57			0.024		0.301		0.174
Cerebellum		0.327		0.024	2.66 ± 2.18			0.059		0.174
Cerebral midline		0.148		0.301		0.059	7.64 ± 5.81			0.232
Cerebrum		0.148		0.174		0.174		0.232	5.69 ± 4.89	

Underlined *P* values indicate significant difference.

### Comparison between paralytic and furious rabies

#### RABV antigen-positive neurons

**Figure 4:**
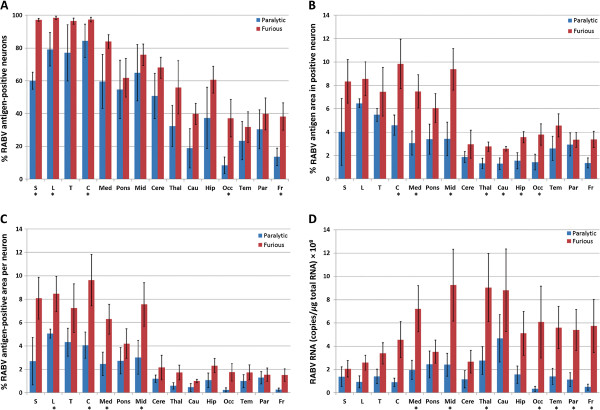
**Rabies viral antigen and RNA pattern comparison between paralytic and furious rabies.** Rabies antigen parameters (**A**-**C**) and RNA levels (**D**) are shown for all CNS regions examined. Blue and red bars refer to paralytic and furious rabies, respectively. S = sacral cord, L = lumbar cord, T = thoracic cord, C = cervical cord, M = medulla oblongata, P = pons, Mb = midbrain, Cb = cerebellum, Th = thalamus, Cd = caudate nucleus, H = hippocampus, O = occipital lobe, Tm = temporal lobe, Pr = parietal lobe, and F = frontal lobe. The viral RNA is expressed as the copy number of rabies/µg total RNA × 10^8^. Asterisks indicate significantly greater RABV antigen or RNA burden in the furious subtype compared to the paralytic subtype.

The percentage of RABV antigen-positive neurons was significantly greater in the furious form compared to the paralytic form in the frontal (*p* = 0.038) and occipital (*p* = 0.029) lobes (Figure 4A). Viral antigen was ubiquitous in the brainstem neurons of both clinical forms with no significant difference. Viral antigen was widespread in the spinal cord of both forms, with a significantly greater percentage noted in the furious form at the cervical (*p* = 0.025), lumbar (*p* = 0.025), and sacral (*p* = 0.025) levels. This quantitative result correlated with the trend noted by semiquantitative assessment (Table 1).

#### RABV antigen area in positive neurons

The area of RABV antigen in positive neurons was greater in the occipital lobe (*p* = 0.038), hippocampus (*p* = 0.023), caudate nucleus (*p* = 0.038), thalamus (*p* = 0.025), midbrain (*p* = 0.014), medulla (*p* = 0.043), and cervical cord (*p* = 0.025) of the furious form compared to the paralytic form (Figure 4B).

#### RABV antigen area per positive neuron

The area of RABV antigen per positive neuron was significantly greater in the occipital lobe (*p* = 0.037), midbrain (*p* = 0.023), medulla (*p* = 0.014), and cervical spinal cord (*p* = 0.024) and lumbar spinal cord (*p* = 0.025) of the furious form compared to the paralytic form (Figure 4C).

#### RABV RNA

Significantly greater levels of viral RNA were demonstrated in all cerebral neocortices (*p* = 0.004, 0.014, 0.038, 0.008, for the frontal, parietal, temporal, and occipital lobes, respectively), thalamus (*p* = 0.024), midbrain (*p* = 0.024), and medulla (*p* = 0.024) in the furious form compared to the paralytic form (Figure 4D). The amount of rabies viral RNA in the spinal cord was comparable in both forms.

#### Pattern of inflammation

More intense inflammation (2 + to 3+) was noted in the brainstem of the paralytic form compared to the furious form (Table 2). The inflammatory reaction in the brain and spinal cord was not remarkable in either form (0 to 1+). CD3-positive T lymphocytes strikingly outnumbered CD20-positive B-cells. The number of T-cells paralleled the inflammatory scores. The density of T-cells (the number of cell/0.0705 mm^2^) at the brainstem was significantly greater in paralytic rabies (Figure 3) compared to the furious subtype (70.7 ± 7.41 vs 13.04 ± 2.43, *p* = 0.004). No significant difference in T cell density was noted between the two clinical forms in other parts of the CNS.

## Discussion

It is intriguing that rabies in humans and dogs can manifest in either furious or paralytic forms. The observation that the same rabid dog transmitted paralytic rabies to one human and furious to another human [21] suggests that host response may play a role in determining the clinical subtype. Previous neuropathological studies in rabies-infected humans have not been able to demonstrate differences between these two clinical subtypes [12]. Immunohistochemistry has been employed in limited studies to detect rabies nucleocapsid antigen, and assessment done in a semi-quantitative fashion. In terms of RABV antigen abundance, the thalamus, basal ganglia, and brainstem have been shown to be preferentially involved over the cerebral hemispheres regardless of the clinical forms [12]. A study in rabid dogs also showed greater RABV antigen burden in the brainstem compared to supratentorial structures [22]. However, in this animal study, only the furious form was included and no spinal cord was available for analysis.

In the present study, semi-quantitative and quantitative assessment of RABV antigen was performed in both furious and paralytic forms at the early stage of the disease, and the spinal cord was studied for the first time in canine rabies. The viral antigen burden in both clinical forms was greatest in the spinal cord, followed by brainstem, and then the brain. This caudal to rostral trend of decreasing RABV antigen abundance was shown by semi-quantitative assessment, and quantitative assessment of all three parameters (% positive neurons, % antigen area in positive neuron, and % antigen area per neuron). Greater viral antigen amount was found in the furious form in most CNS regions including the spinal cord. At first, this latter finding might be viewed as paradoxical since one might expect the viral load in the spinal cord would be greater in the paralytic form. However, it is known that the clinical weakness in the paralytic form is due to peripheral nerve dysfunction, rather than the presence of RABV in the spinal cord [9]. Of diagnostic interest, in one dog with the paralytic form, RABV antigen was found only in the spinal cord. Therefore, for making a diagnosis of rabies in a dog using an antigen detection method, a spinal cord specimen should be included, especially when the suspected animal is sacrificed shortly after the onset of illness or when not yet unconscious from the disease.

RABV RNA was distributed almost uniformly in all CNS regions by the early stage of the disease, regardless of the clinical subtype, reflecting the rapidly aggressive nature of rabies infection. The larger viral RNA load in all CNS sites in the furious form compared to paralytic form may explain the shorter survival period in the furious form and perhaps the more dominant cerebral symptoms seen in both human and canine rabies. Although it has been shown that RABV RNA is synthesized within a cage of nucleocapsid protein in the Negri body-like structures [23], the distribution of viral RNA in our study did not mirror that of rabies nucleocapsid protein. Whereas there were significant differences in the amount of RABV antigen at different levels of the CNS, this was generally not the case for viral RNA. While the brainstem, thalamus, caudate and hippocampus contained higher copy numbers of RABV RNA compared to other regions, this was not statistically significant. The cerebellum was the only site to have significantly less viral RNA compared to the brainstem and only in the furious form. Furthermore, the spinal cord, where RABV antigen was most abundantly found, contained relatively low amounts of viral RNA.

The discrepancy between RABV antigen and RNA amounts reflects the dynamic nature of viral transcription and replication and could be related to differences in production and degradation of RABV mRNA as distinct from viral antigen, and/or host factors controlling of transcription such as miRNAs. Different CNS regions may vary in resistance to viral propagation and cytolytic effects. In support of this hypothesis, rat spinal cord motor neurons are more resistant *in vitro* to RABV-induced cytolysis in a fixed virus model [24]. Other host factors may also play important roles in determining the clinical subtype of rabies infection. A greater immune response occurs with paralytic rabies, as evidenced by cytokine mRNA transcripts in the brain of dogs [18].

A noteworthy finding in our study is that the greatest degree of inflammation was found in the brainstem of dogs with the paralytic form. Similar to other viral infections, T-cells predominated over B-cells in the inflammatory reaction. These findings correlated with the imaging findings showing more abnormal signals by MRI, indicating a greater degree of inflammation, in dogs with the paralytic form compared to dogs with the furious form, particularly at the brainstem level [18]. Moreover, interleukin-1*β* and interferon-*γ* mRNAs were found exclusively in the paralytic form [18]. Our findings also correlate with a mouse model study in which minocycline-treated mice showed reduced inflammation and increased disease severity when infected with SAD-D29, an attenuated strain of rabies [25]. The heightened inflammatory response in the canine brainstem in paralytic rabies is in line with the impairment of neural tract integrity recently demonstrated by increased FLAIR (fluid attenuated inversion recovery) signal on MRI and decreased fractional anisotropy values on diffusion tensor imaging [14]. Disruption of axoplasmic flow by inflammation in the brainstem could potentially retard viral propagation, particularly towards the cerebral hemispheres in the situation of paralytic rabies. This, in turn, could result in a diminished viral load in the brainstem and cerebrum. Why the maximum degree of inflammation was seen in the brainstem is not known. This may reflect the site of initial entry into the CNS by the RABV, since most dogs would be bitten in the neck region, and the virus would then migrate along the cranial nerves to enter the CNS at the level of the brainstem. However, we cannot say with certainty at which sites the dogs in our study were bitten, and thus this remains speculation. We also recognize the small number of animals available for study limits the strength of any conclusions we might reach. Carrying out larger studies in dogs in a controlled laboratory setting would overcome some of these limitations, and would also allow a comparison of clinical and pathologic findings between immune-competent and immune-deficient dogs. However, experimental studies for rabies are generally done in rodents rather than dogs, in part for ethical reasons. There also arise concerns over which virus to use in the laboratory setting. Wild-type virus is difficult to control with respect to the amount of virus to inoculate in order to create a reproducible incubation period, clinical picture, and mortality rate. Laboratory strains of RABV do not duplicate the natural infection. For example, elimination of RABV virulent strains is impeded by apoptosis of migratory inflammatory cells, whereas laboratory RABV strains produce neuronal apoptosis that is not seen with the wild-type virus [22,26-28]. As well, other studies have shown laboratory strains are associated with activation of the host immune response, whereas there is very little response to wild-type virus [29]. Therefore, there are distinct advantages to studying rabies in the natural setting.

Host factors may not be the only explanation for the two clinical subtypes of rabies. We were previously unable to detect any specific differences in the glycoprotein, phosphoprotein and nucleocapsid genes of RABV isolated from the furious and paralytic forms in humans and animals [30]. The glycoprotein gene was found to have minor sequence variations, and cloned sequences of the viral population derived from a single rabies-infected dog showed minor substitutions at both nucleotide and amino acid levels [31]. However, both reports analyzed viruses isolated only from one part of the brain. Hence, it remains possible that viral genetic polymorphisms might play a role in determining the clinical manifestations and influencing the host inflammatory and immune responses.

## Conclusions

Our results provide preliminary evidence that rabies virus appears to have a greater ability for dissemination in the furious subtype compared to the paralytic. The lower amount of rabies viral antigen and RNA in several CNS regions of dogs with the paralytic form is associated with conspicuous inflammation at the brainstem. Different host inflammatory responses might account for the two clinical forms of rabies. This inflammatory reaction could lead to impaired viral dissemination towards the CNS, particularly into the cerebral hemispheres in paralytic rabies, and might explain the longer survival time compared to the furious form. Additional research including controlled laboratory studies is needed to examine this hypothesis.

## Competing interests

The authors declare that they have no competing interests.

## Authors’ contributions

SS designed the study, performed histopathological examination, and participated in data analysis. NT carried out immunohistochemical stainings and image analysis. PP designed computer program for image analysis. AJ performed real-time PCR, and NI performed statistical analysis. VT took care of the animal and collected samples. All authors, including SW, PT, and TH, involved in drafting the manuscript and approved the final version.
